# Multiplex CRISPR/Cas9-based genome engineering enhanced by Drosha-mediated sgRNA-shRNA structure

**DOI:** 10.1038/srep38970

**Published:** 2016-12-12

**Authors:** Qiang Yan, Kun Xu, Jiani Xing, Tingting Zhang, Xin Wang, Zehui Wei, Chonghua Ren, Zhongtian Liu, Simin Shao, Zhiying Zhang

**Affiliations:** 1 College of Animal Science and Technology, Northwest A&F University, Yangling 712100, Shaanxi, China; 2 Institute of applied biology, Shanxi University, 030006, Shanxi, China

## Abstract

The clustered regularly interspaced short palindromic repeats (CRISPR) system has recently been developed into a powerful genome-editing technology, as it requires only two key components (Cas9 protein and sgRNA) to function and further enables multiplex genome targeting and homology-directed repair (HDR) based precise genome editing in a wide variety of organisms. Here, we report a novel and interesting strategy by using the Drosha-mediated sgRNA-shRNA structure to direct Cas9 for multiplex genome targeting and precise genome editing. For multiplex genome targeting assay, we achieved more than 9% simultaneous mutant efficiency for 3 genomic loci among the puromycin-selected cell clones. By introducing the shRNA against DNA ligase IV gene (*LIG4*) into the sgRNA-shRNA construct, the HDR-based precise genome editing efficiency was improved as more than 2-fold. Our works provide a useful tool for multiplex and precise genome modifying in mammalian cells.

The clustered regularly interspaced short palindromic repeats/CRISPR-associated protein (CRISPR/Cas9) technology derived from the bacteria adaptive immune system^1^,^2^,^3^ has been developed as a powerful and versatile platform for genome engineering^4^. It is effective, affordable, scalable and easy to engineer, enabling the induction of site-specific double-strand breaks (DSBs) simply by using Cas9 protein coupled with a single guiding RNA (sgRNA or gRNA)^4^. In addition to these advantages, the natural architecture of the CRISPR locus with arrayed spacers suggests that the CRISPR/Cas9 technology can be used for multiplex genome engineering^1^. To conduct the multiplex targeting in bacterial genome engineering, we artificially assembled the CRISPR structure as we previously reported^5^. Different strategies for delivering set of sgRNAs have been used for mammalian multiplex genome targeting, such as simultaneously using multiple *in vitro* transcribed sgRNAs, multiple sgRNA-expressing cassettes obtained by PCR or multiple sgRNA-expressing plasmids, as well as using single plasmid with multiple sgRNA expression driven by a single or multiple individual promoters. In which, the most direct strategy is delivering *in vitro* transcribed sgRNAs and Cas9 mRNAs into cells by RNA transfection or electroporation, or into embryos by microinjection^6^,^7^. Besides, the direct delivery of Cas9 protein/sgRNA ribonucleoprotein complexes (Cas9 RNPs) have also been reported for high efficient, multiplexed genome engineering^8^. On the other hand, several interesting strategies have been reported with multiple sgRNA expression using a single plasmid vector^9^,^10^,^11^,^12^, which have practically boosted the multiplex genome targeting capability of CRISPR/Cas9 technology.

Short hairpin RNA (shRNA) is an artificial RNA molecule with a tight hairpin turn that can be used to silence the expression of a target gene via RNA interference (RNAi)^13^,^14^. shRNA can be transcribed by either polymerase II or polymerase III depending on the promoter choice. The product mimics pre-microRNA (pre-miRNA) and is known to be processed primarily by Drosha, which is a class II ribonuclease III (RNase III) enzyme^15^, and is the core nuclease that executes the initiation step of miRNA processing in the nucleus^16^. In the light of shRNA processing mechanism, we designed the sgRNA-shRNA structure (U6-sgRNA1-shRNA-sgRNA2), the transcript of which is supposed to be processed by Drosha into functional sgRNAs and shRNAs.

We firstly applied the DsRed-eGFP surrogate reporter as we previously reported^17^ to confirm the activities of the sgRNAs transcribed from the sgRNA-shRNA structure. The *eGFP* reporter gene in the DsRed-eGFP surrogate reporter is designed to be interrupted by the sgRNA target, and to be repaired precisely through the single strand annealing (SSA) pathway when DSBs are induced by sgRNA/Cas9. Thus, the sgRNA activity for directing Cas9 nuclease function can be evaluated indirectly by the fluorescent measurement. SSA is a special DNA repair mechanism, which can promote recombination between tandem repeated DNA sequences and induce the deletion of intervening fragments^18^,^19^. Although it has been well accepted that in mammalian cells DSBs are primarily repaired by the non-homologous end joining (NHEJ) repair mechanism^20^,^21^, SSA may be more frequent if substantial repetitive elements are proximal to the break ends^22^. As previously reported, we have developed a series of surrogate reporters based on the SSA-mediated precise repair of reporter genes for evaluating the activity of artificial site-specific nucleases or enriching genetically modified cells^17^,^23^,^24^.

Secondly, we tried to apply the sgRNA-shRNA structure harboring more sgRNAs for multiplex genome targeting. To improve the targeting efficiency, we used the same RPG (DsRed-Puro^r^-eGFP) surrogate reporter system as previously reported^17^,^23^ to aid the enrichment of positive cells with genomic modification as well as the selection of positive cell clones. When the nuclease functions on the RPG reporter, the interrupted puromycin resistant gene (*Puro*^*r*^) can be repaired through the SSA repair pathway precisely. The functional *Puro*^*r*^ gene expression represents that the nuclease (sgRNA/Cas9) is positive, and allows the enrichment of cells with functional sgRNA/Cas9 via puromycin selection. Thus, as a result, the genome modification in these cells can be increased dramatically compared with unselected cells. Besides, the DsRed and eGFP expression allows for the visualization of the transfection efficiency and the sgRNA activity. When the sgRNA/Cas9 complex has functioned on genome DNA, DSBs induced are primarily repaired by the NHEJ repair pathway, typically causing small insertions or deletions (indels). Hence, the indel frequency is usually used for sgRNA/Cas9 and other nucleases activity measurement.

Besides, precise genome editing, such as codon replacement, requires the strategy dependent on the recombination of donor DNA and targeted genomic DNA mediated by the homology-directed repair (HDR) pathway^25^. It has been reported that the efficiency of HDR-based precise genome editing can be boosted by the transient inhibition of NHEJ key molecules, such as DNA ligase IV (LIG4) and KU70^26^,^27^. As shRNA is coupled with the sgRNA expression, the sgRNA-shRNA structure was supposed to be capable to facilitate the precise genome editing by using functional shRNA. We next introduced the shRNA against *LIG4* gene into the sgRNA-shRNA construct as an example to verify this capability.

Briefly, in this study we developed a novel and interesting Drosha-mediated sgRNA-shRNA structure combining the expression of sgRNA and shRNA, from which sgRNAs could be used to direct Cas9 for multiplex genome targeting meanwhile shRNAs enhancing the precise genome editing by down regulating *LIG4* through RNAi.

## Results

### The strategy for combining sgRNA and shRNA transcripts

**Figure 1:**
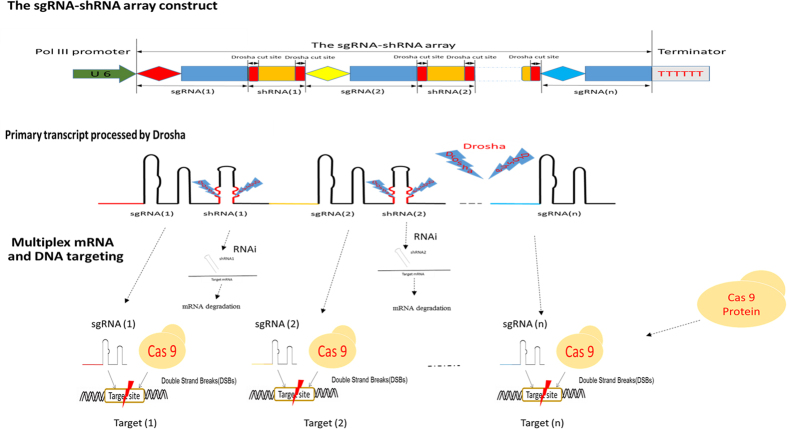
The schematic of the sgRNA-shRNA structure combining sgRNA and shRNA transcripts. The sgRNA-shRNA structure is designed to be driven by the U6 promoter, and the primary transcript is supposed to be processed into individual sgRNAs and shRNAs by the endogenous Drosha in the nucleus. The sgRNAs can direct the Cas9 protein for multiplex genome DNA targeting, and the siRNAs can be applied for RNA interference (RNAi).

In this study, we hypothesized that multiple sgRNAs and shRNAs can be assembled in a sgRNA-shRNA structure format with interval sequences of the Drosha cutting site (Fig. 1). The sgRNA-shRNA structure can be driven by a single polymerase III U6 promoter. Actually, we have also tried polymerase II CMV, EF1α and CAG promoters, but all of them failed because of the low transcribing efficiency (data not shown). The sgRNA-shRNA transcript was supposed to be processed into functional sgRNAs and shRNAs by endogenous Drosha. The multiple sgRNAs can be used to direct Cas9 protein for multiplex genome targeting. Moreover, by introducing the shRNA against *LIG4* gene as described above, the sgRNA-shRNA construct can be applied to enhance the HDR-based genome editing. The processing of Drosha may leave some unnecessary nucleotide sequences at the 3′ and/or 5′ ends of the cleaved sgRNAs and shRNAs. It has been reported that deletions from the 5′ end of a sgRNA do have significant effect on its function. But, it argues for the additions. Our previous publication has reported that additions at the 5′ end of a sgRNA didn’t reduce its activity obviously^17^. There is another report talking about the HDV-sgRNA structure claimed that even there was a long self-cleaving hepatitis delta virus (HDV) ribozyme sequence at the 5′ end, the sgRNA still worked very well^11^. Besides, the applications of the Csy4-sgRNA structure suggested the sgRNAs with redundant Csy4 sequence at the 3′ end also functioned well^12^,^28^. On the other hand, shRNAs with some unnecessary nucleotides at the ends also have been reported still with bioactivities^31^.

### Verifying the activities of sgRNAs transcribed from the sgRNA-shRNA structure

**Figure 2:**
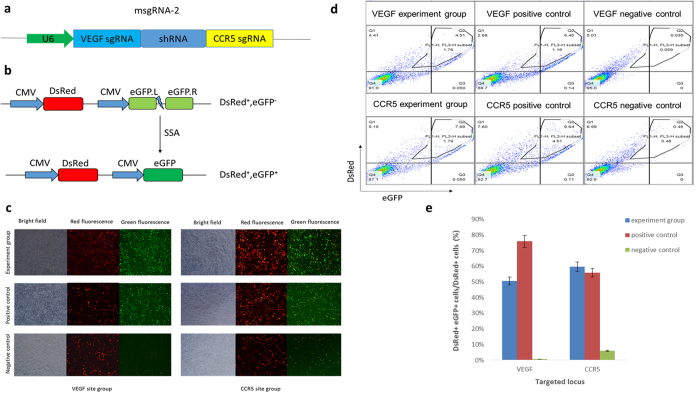
Surrogate reporter assay verifying the activities of sgRNAs transcribed from the sgRNA-shRNA structure. (**a**) The design of the msgRNA-2 construct. An irrelevant shRNA flanked with the Drosha cutting sites was designed and cloned between VEGF.sgRNA and CCR5.sgRNA scaffolds in the msgRNA-2 plasmid. (**b**) The illustration of the DsRed-eGFP surrogate reporter construct based on SSA repair. The *DsRed* marker gene and *eGFP* reporter gene were designed to be driven by individual CMV promoters. The *eGFP* reporter gene was interrupted by the sgRNA target sequence flanked with direct repeats as SSA arms. Once the sgRNA/Cas9 complex functioned on the target in the reporter, the *eGFP* reporter gene would be restored through the SSA repair pathway. Thus, the sgRNA activity for directing Cas9 nuclease function can be evaluated indirectly by the fluorescent measurement. (**c**) Representative visualization of DsRed and eGFP expression by fluorescence microscopy. HEK293T cells were co-transfected with different plasmid groups (Supplementary Table 1). At least three independent replicates were performed for each transfection. The cells were observed and photographed with a fluorescence microscope 2 days after the transfection. (**d**) Flow cytometric counting results for the DsRed^+^ and DsRed^+^ eGFP^+^ cells. 30000 cells were counted to identify the DsRed^+^ and DsRed^+^ eGFP^+^ positive cells. (**e**) Comparison of the activities of sgRNAs from the sgRNA-shRNA structure with the single sgRNA positive controls. The percentage of DsRed^+^eGFP^+^ positive cells compared with DsRed^+^ cells was calculated as an indirect measurement to evaluate the sgRNA activities for directing Cas9 for targeting. Error bars represent standard deviation, n = 3.

To validate that the sgRNA-shRNA transcript can be processed to functional sgRNAs, we generated the msgRNA-2 construct containing VEGF.sgRNA, CCR5.sgRNA and an irrelevant shRNA^32^ (Fig. 2a, Supplementary Fig.1a and Sequence 1) and the corresponding DsRed-eGFP surrogate reporter vectors (Fig. 2b) for these two sgRNAs as we previously reported^17^. Human embryonic kidney 293 T cells were co-transfected with the msgRNA-2 plasmid, the DsRed-eGFP surrogate reporter (pRG) and the hStCas9 expression vector^17^, and parallel transfections were done for related positive and negative groups. The transfection efficiency and sgRNA activity were visualized using the red and green fluorescence (Fig. 2c). DsRed^+^ and DsRed^+^eGFP^+^ positive cells were counted by flow cytometer (Fig. 2d). The percentage of DsRed^+^eGFP^+^ positive cells compared with DsRed^+^ cells was calculated as an indirect measurement to evaluate the sgRNA activities. The results demonstrated that the activities for VEGF.sgRNA and CCR5.sgRNA were about 51% and 60%, respectively. When compared with the positive single sgRNA controls whose activities were76% and 56% respectively, there was a little bit increase for CCR5.sgRNA and about one third decrease for VEGF.sgRNA (Fig. 2e). Nevertheless, the results suggested that the sgRNA-shRNA structure can be transcribed and processed in cells to generate functional sgRNAs.

### Multiplex genome targeting applying the sgRNA-shRNA structure

**Figure 3:**
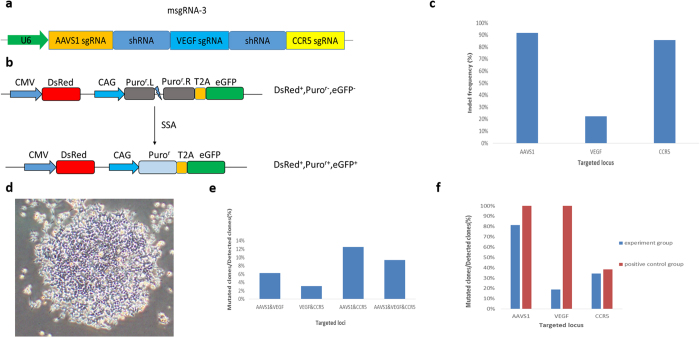
Multiplex genome targeting assay applying the sgRNA-shRNA structure. (**a**) The design of the msgRNA-3 construct. The AAVS1.sgRNA scaffold and another irrelevant shRNA were inserted into the msgRNA-2 construct upstream of the VEGF.sgRNA scaffold. (**b**) The illustration of the RPG surrogate reporter construct based on SSA repair. Similar with the DsRed-eGFP surrogate reporter, when the nuclease functions on the RPG reporter, the interrupted puromycin resistant reporter gene (*Puro*^*r*^) could be repaired through the SSA repair pathway precisely, facilitating the enrichment of genome edited cells or the screening cell clones by puromycin selection. (**c**) The indel frequencies within the cell pool after the enrichment of puromycin selection. The genome DNA of the cell pool was isolated and used as template for PCR amplification of these loci. The PCR products were routinely cloned by T-A cloning technique generating the “T-A clones” for further sequencing analysis (Supplementary Fig. 2). (**d**) Representative cell clones screened by puromycin selection. Dozens of cell clones were screened for each group. Genome DNAs for these cell clones were isolated. PCR and sequencing analysis of the three genome loci (AAVS1, VEGF and CCR5) were conducted to identify the mutants. The results with lapped peaks around the target loci were considered to be positive mutated clones. (**e**) The overall percentages of diplex and triplex targeted positive clones within detected clones. Three replicates were designed for each transfection and single clones were collected after puromycin screening. Generally, 12, 10 and 10 clones were analyzed for the three experimental groups, and the total triplex targeting efficiency was more than 9% (3/32, Supplementary Table 3). Diplex targeting meant two simultaneous targeting occurred, and the third target site was not cleaved. (**f**) The overall percentages of mutated clones for each targeted locus within detected clones (Supplementary Table 4). For the msgRNA-3 experiment groups, the mutants for each locus within the total 32 detected clones were statistically analyzed. For the three single sgRNA positive controls, cell clones were also screened by puromycin selection, and the mutant analyses were conducted individually with about 10 cell clones analyzed by sequencing for identifying the mutants.

To further apply the sgRNA-shRNA structure for multiplex genomic DNA targeting, the msgRNA-3 construct was generated by inserting the AAVS1.sgRNA and another irrelevant shRNA into the msgRNA-2 construct upstream of the VEGF.sgRNA (Fig. 3a, Supplementary Fig. 1b and Sequence2). The corresponding RPG surrogate reporter vectors (pRPG, Fig. 3b) were constructed as we previously reported^17^,^23^ and used to facilitate the genome targeting assays. The HEK293T cells were preliminarily co-transfected with the msgRNA-3 construct, hStCas9 expression vectors together with the pRPG.AAVS1 vector. The *DsRed* marker gene and the *eGFP* reporter gene within the RPG surrogate reporter were used to evaluate the transfection efficiency and the AAVS1.sgRNA activity by fluorescent visualization. The *Puro*^*r*^gene once been repaired could facilitate the enrichment of genome edited cells by puromycin selection as described. To identify the indels at these three msgRNA3/Cas9-targeted loci (AAVS1, VEGF and CCR5) generated routinely by the NHEJ repair pathway, the cells as a pool were collected after the transfection and puromycin selection, and the genome DNA was isolated and used as template for PCR amplification. The PCR products were routinely cloned by T-A cloning technique into the T vector (pGEM^®^-T) generating the “T-A clones” for further sequencing analysis. Sequencing results of the “T-A clones” for the AAVS1, VEGF and CCR5 target loci demonstrated approximately 92%, 22% and 86% indel frequencies (Fig. 3c, Supplementary Fig. 2), respectively. The result for VEGF locus dramatically lower than the other two loci may be caused by the middle effect of the VEGF.sgRNA. Whether it is induced by the insufficient cleavage of the sgRNA-shRNA transcript or other reasons remains to be investigated.

Next, we asked whether the multiplex genome targeting did occur within a single cell. Hence, we conducted further experiments generating cell clones by puromycin selection for detailed detection. Considering different RPG reporters may produce different biases in their results, we conducted 3 parallel experiments respectively using RPG surrogate reporters for AAVS1.sgRNA, VEGF.sgRNA and CCR5.sgRNA (Supplementary Table 2). Moreover, to address the middle effect as we observed above, we added single sgRNA as positive controls. Dozens of cell clones (Fig. 3d) were screened by puromycin selection for each group. Genome DNA for these cell clones were prepared. PCR and sequencing analysis of the three genome loci were conducted to identify triplex targeted positive cell clones. Generally, 12, 10 and 10 clones were analyzed for these three experimental groups, and the total triplex targeting efficiency was more than 9% (3/32). Moreover, we also successfully identified diplex targeted positive clones with AAVS1 and VEGF, VEGF and CCR5, AAVS1 and CCR5 simultaneous mutations (Fig. 3e, Supplementary Table 3). The overall mutation frequencies within the detected 32 clones for the AAVS1, VEGF and CCR5 sites were 81%, 19% and 34%, respectively (Fig. 3f, Supplementary Table 4). By comparing the three experimental groups, we found no obvious bias effect for using different RPG surrogate reporters. On the other hand, the previously mentioned middle effect still did exist when compared with the single sgRNA positive controls (Fig. 3f, Supplementary Table 4).

### Enhancing precise genome editing with shRNA of the sgRNA-shRNA structure

**Figure 4:**
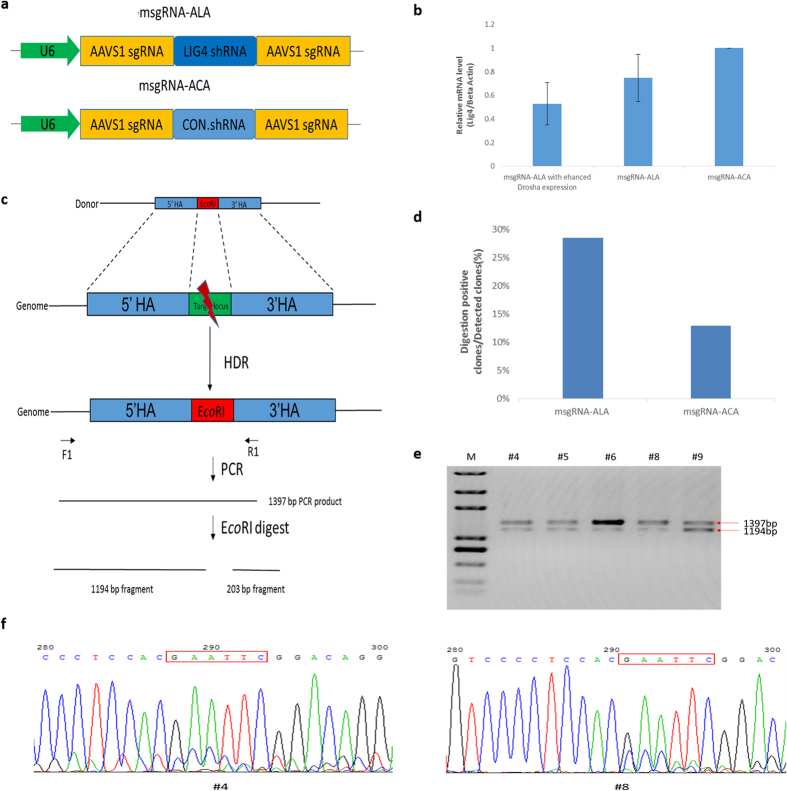
HDR-based precise genome editing assay applying the sgRNA-shRNA structure. (**a**) The design of the msgRNA-ALA construct and the control msgRNA-ACA. The LIG4.shRNA and CON.shRNA represent the shRNA designed against *LIG4* gene and the control irrelevant shRNA respectively. The two AAVS1.sgRNA scaffolds on the both side of shRNA were designed targeting the same site. (**b**) The down-regulation of *LIG4* gene with msgRNA-ALA. The relative LIG4 mRNA levels (LIG4/Beta-actin) were measured using qRT-PCR. Error bars represent standard deviation, n = 3. (**c**) An illustration for the HDR-based genome editing of AAVS1 locus and the detection for positive clones. The donor DNA was designed with the PAM motif of the AAVS1 target site replaced by the *Eco*R I cutting site. This design allows to identify genome edited cell clones by PCR and *Eco*R I-digesting assay. 1194 bp and 203 bp bands should appear if the genome DNA has been successfully edited by HDR-based repair. (**d**) Percentage of digestion positive clones within detected clones was calculated to evaluate the precise genome editing efficiency. The PCR and *Eco*R I-digesting assay demonstrated 10 positive clones out of 35 cell clones (10/35) for the msgRNA-ALA group, while 4 out of 31 clones (4/31) for the msgRNA-ACA control (Supplementary Fig. 3). Three independent replicates were performed for each group. (**e**) The representative results for the detection of positive clones by *Eco*R I digestion. The two bands (1397 bp and 1194 bp) indicated that the detected clones were all heterozygotes with one allele edited. This was a cropped gel and the full-length gel images were shown in Supplementary Fig. 4. (**f**) Representative sequencing results for the PCR products from clones #4 and #8. The lapped peaks indicated that both clones were heterozygotes. The *Eco*R I site that was introduced is indicated by red rectangles.

Taking the advantage of the shRNA expression coupled with sgRNA, we supposed that the sgRNA-shRNA structure could be used to facilitate the HDR-based precise genome editing by introducing functional shRNA, such as the shRNA against *LIG4* gene. Hence, we generated the msgRNA-ALA construct (AAVS1.sgRNA-LIG4.shRNA-AAVS1.sgRNA) and the control msgRNA-ACA construct with irrelevant shRNA (AAVS1.sgRNA-CON.shRNA-AAVS1.sgRNA) (Fig. 4a). To confirm the down-regulation of *LIG4* gene in the cells transfected with msgRNA-ALA plasmid, the quantitative RT-PCR analysis was performed and the result turned out that the relative *LIG4* expression was declined about 25% compared with the control (Fig. 4b). Moreover, When an exogenous vector pFRT-TODestFLAGHAhFMRPiso1^33^,^34^, purchased from Addgene (Addgene Plasmid #48690) with Drosha expression enhancing ability, was co-transfected with msgRNA-ALA plasmid, the relative *LIG4* expression declined to about 50% (Fig. 4b),

To conduct the precise genome editing assay, the donor DNA was designed with the PAM of the AAVS1 target site replaced by the *Eco*R I cutting site and was generated by overlap PCR. This design allows to identify genome edited cell clones by PCR and *Eco*R I-digesting assay (Fig. 4c). HEK293T cells were transfected with the msgRNA-ALA or msgRNA-ACA plasmid, the surrogate reporter pRPG.AAVS1, hStCas9 expression vector and the donor DNA. Cell clones were screened by puromycin selection. The corresponding genome DNA was extracted for the PCR and *Eco*R I-digesting assay. The PCR product was supposed to be 1397 bp in length and could be digested into two fragments (1194 bp and 203 bp) if the genome DNA has been successfully edited by HDR-based repair. Percentage of digestion positive clones within total clones was calculated to evaluate the precise genome editing efficiency. The results demonstrated that the frequency for msgRNA-ALA construct was 29% (10/35), which was more than 1-fold higher than the msgRNA-ACA control group (13%, 4/31) (Fig. 4d, Supplementary Fig. 3). The PCR products were supposed to be partially digested if the clone was heterozygous with only one allele edited. Unfortunately, we detected no double-allele precise editing within these detected clones (Fig. 4e). Nevertheless, further sequencing analysis of representative clones confirmed the editing (Fig. 4f).

## Discussion

In this study, we developed a novel and interesting sgRNA-shRNA structure for combining the sgRNA and shRNA transcript, which was supposed to be processed by endogenous Drosha into functional sgRNAs and shRNAs. We firstly tried the polymerase II CMV, EF1α and CAG promoters for driving the sgRNA-shRNA transcript, but all failed when verifying the sgRNA activity in the DsRed-eGFP surrogate reporter assay. Next, we succeeded with the polymerase III U6 promoter, suggesting the U6 promoter was much stronger than polymerase II promoters for driving the sgRNA-shRNA transcript with complicated hairpin structures.

The sgRNA-shRNA structure was designed mimicking the shRNA-processing mechanism and the transcript was supposed to be processed into functional sgRNAs and shRNAs by endogenous Drosha. However, the processing of Drosha may leave some unnecessary nucleotide sequences at the ends of the cleaved RNAs. Although we have proved that the sgRNAs and shRNAs transcribed from the sgRNA-shRNA structure did function with bioactivities, it remains to be investigated for further addressing how this structure is processed, what ends for the RNAs look like and what exact effects on their bioactivities.

We secondly applied the sgRNA-shRNA structure for multiplex genome engineering. To facilitate the genome targeting assay, we used the RPG surrogate reporter as we previously reported, which allows the enrichment of genome edited cells by puromycin selection^17^,^23^. As reported^17^,^23^, in the absence of puromycin selection, the indel frequencies are very low, which we thought was caused by the limitation of the transfection efficiency of multiple plasmids and the rapid growth of HEK293T cells. Similar publications using mRFP/eGFP dual-fluorescence surrogate reporter for the enrichment through flow cytometric method also has been reported^35^. Besides, a lot of publications also used the selecting marker gene (such as *Neo*^*r*^) or fluorescence gene (such as *eGFP*) in the sgRNA/Cas9 expression vector for “transfected cells” enrichment. Surprisingly, it has been reported that the direct delivery of Cas9 RNPs without selection could achieve three-locus indel frequency up to 65%^8^. Although we achieved generating the triplex targeted cell clones as well, the efficiency was much lower (some more than 9%). The reliance on puromycin selection appeared to limit the usefulness of our system. However, the puromycin selection may help with the quick generation of positive cell clones.

During the multiplex genome targeting assay, we observed the middle effect with dramatically decrease of VEGF.sgRNA activity (Fig. 3c,f), which may be the limiting factor for the relatively low triplex genome targeting efficiency (just a little bit more than 9%). By now it’s not clear yet what the ends look like after Drosha processing, possibly the additions to the 5′ or 3′ ends of the gRNA would significantly affect genome editing activity. To solve this problem, we can try several strategies in the future study. First, simply enhancing Drosha expression by co-transfecting with an exogenous plasmid may be a simple way, as our primary experiments showed the RNAi efficiency of LIG4 shRNA was improved (Fig. 4b). Second, we may optimize the Drosha cutting site for more efficient cleavage of the sgRNA-shRNA transcript and less remaining residues after the processing. Moreover, other members from RNase III superfamily can be explored. Additionally, the byproduct shRNA can be utilized to inhibit the factors that function against Drosha cleavage activity or the NHEJ pathway. Finally, in our design the restriction enzyme sites were used in the front of the sgRNA sequences to clone the sgRNA-shRNA structure, which may also leave residues affecting the sgRNA activity. An alternative seamless assembly strategy for generating the structure can be applied as we previously reported^36^.

One character of our structure is that the sgRNA-shRNA can be assembled in an array format driven by a single U6 promoter. The sgRNA-shRNA transcript is supposed to be processed into functional RNAs through the endogenous shRNA-processing mechanism. Several similar systems have been developed. The tRNA-sgRNA structure used in rice plant^10^ and the HDV-sgRNA structure used in industrial yeast^11^ were both reported to be driven by polymerase III promoters, which utilizing endogenous tRNA-processing mechanism for multiple sgRNAs maturation and boosting CRISPR/Cas9 multiplex genome targeting capability. However, so far we have found no applications of these two structures in mammalian species. There is another Csy4-sgRNA structure which has been reported to be driven also by a single U6 promoter capable for double active sgRNAs expression in human cells^12^. The cleavage of the Csy4-sgRNA transcript relies on exogenous Csy4 RNase of *Pseudomonas aeruginosa*. The Csy4-mediated multiple genome editing was efficient^12^,^28^. but scientists have found that as an exogenous protein Csy4 was cytotoxic^28^ in the transfected cells and also harmful to the injected zebrafish embryos during their development^29^. By contrast, Drosha is an endogenous protein without cytotoxicity. In general, our system give an alternative option for multiple genome targeting research except for the tools as we mentioned above. The significant advantage of our sgRNA-shRNA structure is the combination of shRNA and sgRNA expression. The transcribed sgRNAs can be used to direct Cas9 protein for genome targeting, while the byproduct shRNA can be used to silence related functional genes. As it has been reported that silencing *KU70* and *LIG4* gene could promote the efficiency of CRISPR/Cas9-mediated HDR by 4~5-fold^26^, we supposed that the sgRNA-shRNA structure could be used to enhance the HDR-based precise genome editing. By introducing the shRNA against *LIG4* gene into the sgRNA-shRNA construct, we achieved the efficiency of precise genome editing more than 2-fold of the control. However, the result turned out to be some lower than we expected. As the expression of *LIG4* gene was only declined about 25% and could be further decreased about 50% after Drosha was higher expressed (Fig. 4b), we speculated that the processing of the shRNA was also insufficient just like the middle effect as we observed and could be promoted. That’s why we are trying to use this strategy to improve sgRNA-shRNA maturation process and HDR mediated precise genome editing efficiency.

In conclusion, we developed a novel sgRNA-shRNA structure for combining the sgRNA and shRNA transcript. The sgRNA-shRNA transcript could be driven by a single U6 promoter and was designed to be processed by endogenous Drosha into functional sgRNAs and shRNAs. The sgRNA-shRNA construct can be applied for both multiplex genome targeting and precise genome editing and is expected to facilitate more sophisticated Cas9 applications.

## Methods

### Preparation of plasmid vectors and HDR donor DNA

The CRISPR/Cas9 system used in this study was prepared as we previously described^17^. Briefly, the hStCas9 expression vector (pll3.7-mU6-CMV-hStCas9) was constructed previously with the humanized *S. thermophilus Cas9* (hStCas9) driven by the CMV promoter and no sgRNA scaffold cloned^17^. The sgRNA target sites used for VEGF, CCR5 and AAVS1 loci were CTCGGCCACCACAGGGAAGCTGG, CACACTTGTCACCACCCCAAAGGTG and CTGTCCCCTCCACCCCACAGTGG, respectively. The corresponding sgRNA scaffolds were cloned into pll3.7-mU6-CMV-hStCas9 vector after the mU6 promoter to generate the control single sgRNA expressing vectors. The DsRed-eGFP surrogate reporters^17^ (pRG.VEGF and pRG.CCR5) and the DsRed-Puro^r^-eGFP (RPG) surrogate reporters^17^,^23^ (pRPG.VEGF, pRPG.CCR5 and pRPG.AAVS1) were cloned by oligonucleotides-annealing as previously reported.

The sgRNA-shRNA construct vectors msgRNA-2, msgRNA-3, msgRNA-ALA and msgRNA-ACA were constructed using standard cloning methods with pcDNA3.1(+) as the backbone plasmid. In brief, the sgRNA-shRNA cassettes for these vectors were VEGF.sgRNA-CON.shRNA-CCR5.sgRNA (Fig. 2a, Supplementary Sequence 1), AAVS1.sgRNA-CON.shRNA-VEGF.sgRNA-CON.shRNA-CCR5.sgRNA (Fig. 3a, Supplementary Sequence 2), AAVS1.sgRNA-LIG4.shRNA-AAVS1.sgRNA and AAVS1.sgRNA-CON.shRNA-AAVS1.sgRNA (Fig. 4a,), respectively. The CON.shRNA represented the control irrelevant shRNA as we previously used for mouse CD40 mRNA interference^32^. The LIG4.shRNA represented the shRNA designed against *LIG4* gene. These sgRNA-shRNA cassettes were cloned into the eukaryotic expression vector pcDNA3.1(+) with the CMV promoter replaced by the hU6 promoter (Supplementary Fig. 1).

The HDR donor DNA for AAVS1 locus editing was generated by overlap PCR with the PAM motif of AAVS1 target site replaced by the *Eco*R I cutting site, which allows the detection of positive clones by PCR and an *Eco*R I-digesting assay (Fig. 4c). The 5′ and 3′ homologous arms (HA) were designed to be ~1.0 kb in length.

### Cell culture

The human embryonic kidney 293 T cells used in this study were maintained in DMEM supplemented with 100 U/mL of penicillin, 100 μg/mL of streptomycin and 10% (v/v) FBS, at 37 °C and 5% CO_2_.

### DsRed-eGFP surrogate reporter assay

HEK293T cells were co-transfected with different plasmid groups (Supplementary Table 1, a total of 1.6 μg plasmid DNA per transfection) in 24-well plates using Sofast transfection reagent (Xiamen, Sunma Biotechnology Co., Ltd. China) according to the user manual. Three independent replicates were performed for each transfection. The cells were observed and photographed with a fluorescence microscope 2 days after the transfection. The transfection efficiency and sgRNA activity were visualized preliminarily using the red and green fluorescence. Then, the cells were harvested and subjected to flow cytometric analysis. 30000 cells were counted to identify the DsRed^+^ and DsRed^+^ eGFP^+^ positive cells. The percentage of DsRed^+^eGFP^+^ positive cells compared with DsRed^+^ cells was calculated as an indirect measurement to evaluate the sgRNA activities for directing Cas9 for targeting.

### Multiplex genome targeting assays

For preliminary multiplex genome targeting, HEK293T cells were co-transfected with the msgRNA-3 plasmid, pRPG.AAVS1 and hStCas9 expression vector (pll3.7-mU6-CMV-hStCas9). 2 days after transfection, the medium was supplemented with puromycin at a final concentration of 3 μg /mL and changed daily. 2 days after the puromycin treatment, the cell pool was collected and the genome DNA was isolated using genome DNA isolating kit (Omega) by standard procedure. PCR amplification was performed using the isolated genome DNA as template, and the PCR products for AAVS1, VEGF and CCR5 target loci were routinely cloned into pGEM^®^-T Easy vector by T-A cloning technique according to the manual of pGEM^®^-T Easy Vector System I (Promega), generating the “T-A clones” for sequencing to check the indels.

To investigate whether multiplex genome targeting can be achieved in a single cell, we conducted further experiment screening cell clones for detailed detection. HEK293T cells were co-transfected with different plasmid groups in 12-well plates (Supplementary Table 2, with a total of 3.2 μg plasmid DNA per transfection.). 2 days after transfection, the medium was supplemented with puromycin and changed daily. 8 days after the puromycin treatment, the selected cell colonies were picked and the dilutions were seeded in 48-well plates for proliferation. The cell clones were further cultured in 12-well plates for genome DNA preparation. The PCR products for the AAVS1, VEGF and CCR5 target loci were sequenced and the results with lapped peaks around the target loci were considered to be positive mutated clones. Three independent replicates were performed for each transfection.

### Quantitative RT-PCR assay

To confirm the down-regulation of *LIG4* gene by msgRNA-ALA, HEK293T cells were transfected by msgRNA-ALA plasmid together with purchased pFRT-TODestFLAGHAhFMRPiso1 or alone. Another group was transfected with msgRNA-ACA as control. 2 days after the transfection, the cells were harvest and corresponding total RNAs were isolated using RNAiso Plus (Takara) according to the manufacturer’s instructions. A total of 1 μg of RNA was reverse transcribed into first-strand cDNA with RT primer mix using the PrimeScript™RT reagent Kit with gDNA Eraser (Takara). Realtime PCR was performed using SYBR^®^ Premix Ex Taq II (Tli RNaseH Plus) (Takara) and CFX96 Touch™ Real-Time PCR Detection System (Bio-Rad) following the manufacturer’s protocol. The housekeeping *Beta actin* gene was used as the internal control for normalization. The primers used in the quantitative RT-PCR assay were listed in Supplementary Table 5. Three replicated transfections for this part were performed and the result were analyzed using the 2^−ΔΔCT^ method.

### HDR-based precise genome editing assay

The transfection was performed with msgRNA-ALA or msgRNA-ACA plasmid, pRPG.AAVS1 reporter, the hStCas9 expression vector (a total of 3.2 μg plasmid DNA per transfection, molar ratio = 1.5:0.8:1) and PCR product of HDR donor DNA (80 ng per transfection) in 12-well plates. The cell clones were screened and cultured as described above. Three replicated transfection were performed. The corresponding genomic DNAs were extracted for PCR and the *Eco*R I-digesting assay. The PCR product was designed to be 1397 bp in length and would be digested by *Eco*R I into two fragments (1194 bp and 203 bp) if the corresponding genome DNA was successfully edited by HDR-based repair. The PCR product was partially digested if the clone was heterozygous with only one allele edited. Percentage of digestion positive clones within detected clones (Supplementary Fig. 3) was calculated to evaluate the precise genome editing efficiency. Sequencing analysis of the PCR products from the representative positive clones was also performed to further confirm the editing.

## Additional Information

**How to cite this article**: Yan, Q. *et al*. Multiplex CRISPR/Cas9-based genome engineering enhanced by Drosha-mediated sgRNA-shRNA structure. *Sci. Rep.* **6**, 38970; doi: 10.1038/srep38970 (2016).

**Publisher's note:** Springer Nature remains neutral with regard to jurisdictional claims in published maps and institutional affiliations.

## Supplementary Material

Supplementary Information
