# Supplementary material for: Multiplex CRISPR/Cas9-based genome engineering enhanced by Drosha-mediated sgRNA-shRNA structure
Source: Sci Rep. 2016 Dec 12;6:38970. doi: 10.1038/srep38970 (PMC5150520; doi:10.1038/srep38970)
Supplement: Supplementary Information [file srep38970-s1.pdf]

Multiplex CRISPR/Cas9-based genome engineering enhanced by Drosha-mediated sgRNA-shRNA structure

Qiang Yan[#1], Kun Xu[#1], Jiani Xing[1], Tingting Zhang[2], Xin Wang[1], Zehui Wei[1], Chonghua Ren[1], Zhongtian Liu[1], Simin Shao[1], Zhiying Zhang*[1]

[1] College of Animal Science and Technology, Northwest A&F University, Yangling 712100, Shaanxi, China

[2] Institute of applied biology, Shanxi University, 030006, Shanxi, China

[#] These authors contributed equally to this work.

*To whom correspondence should be addressed. Tel: 029-87092120.

Fax: 029-87092164. Email: zhangzhy@nwsuaf.edu.cn.

Supplementary Information

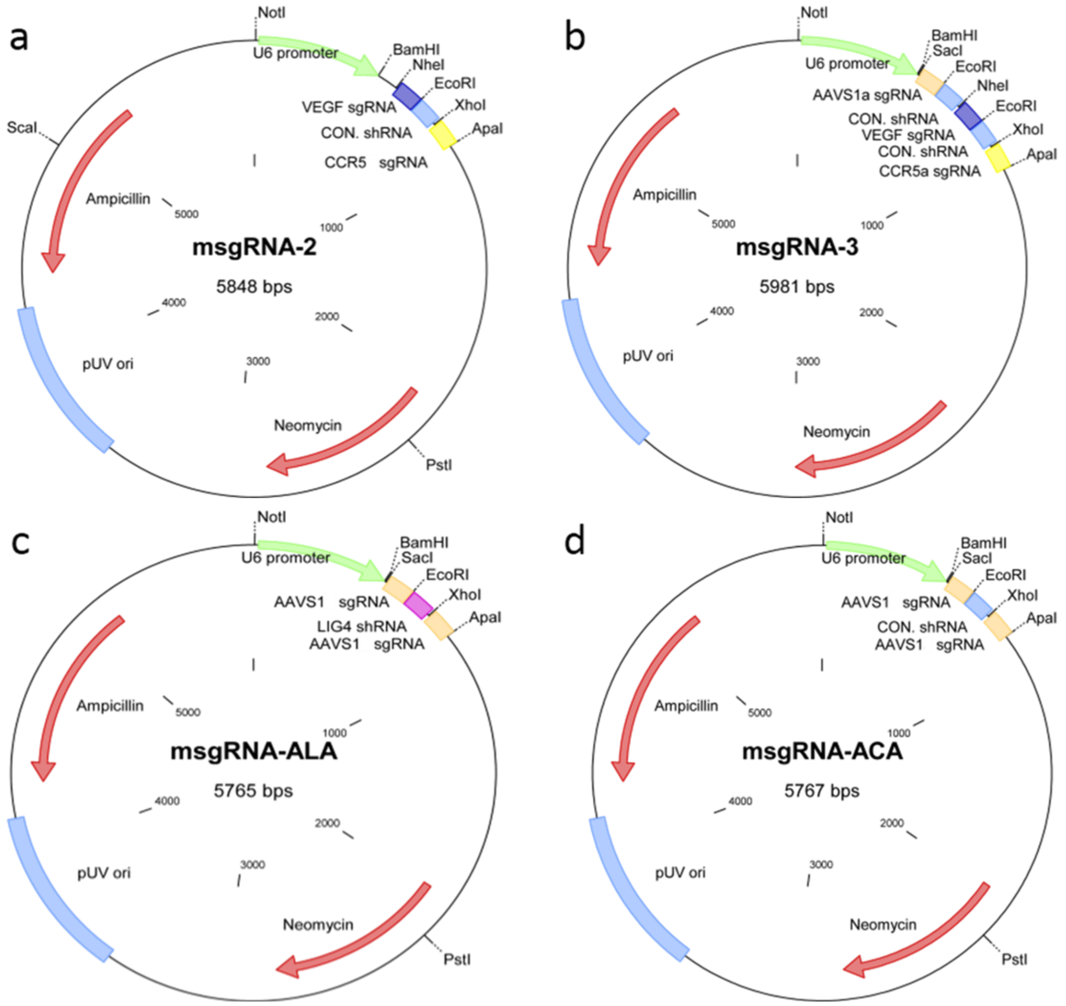

Supplementary Figure 1 Plasmid maps for the constructs: (a) msgRNA-2, (b) msgRNA-3, (c) msgRNA-ALA and (d) msgRNA-ACA

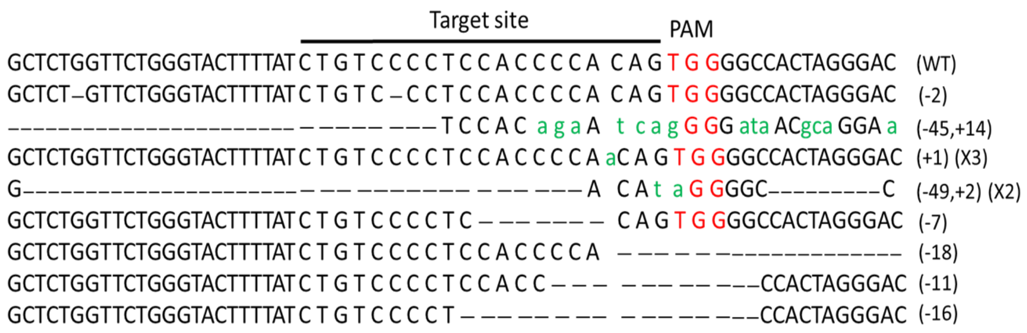

(a) AAVS1 site (11/12, 91.67%)

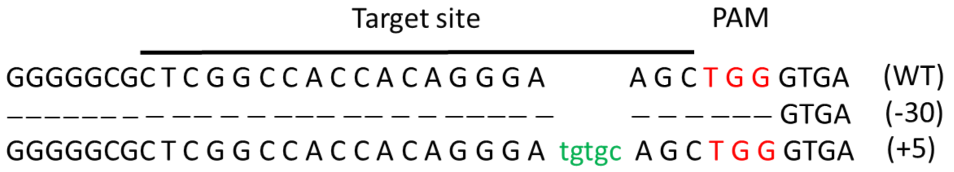

(b) VEGF site (2/9, 22.22%)

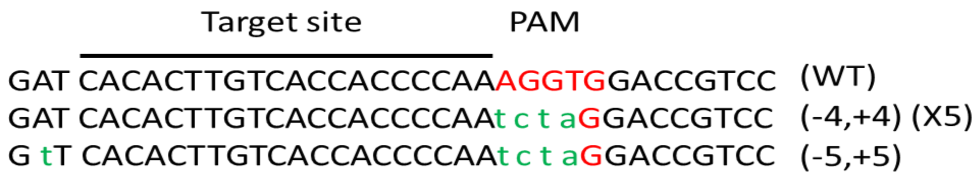

(c) CCR5 site (6/7, 85.71%)

Supplementary Figure 2 The indels within the detected "T-A clones" from the cell pool targeted by msgRNA-3. (a) AAVS1 site, (b) VEGF site, (c) CCR5 site

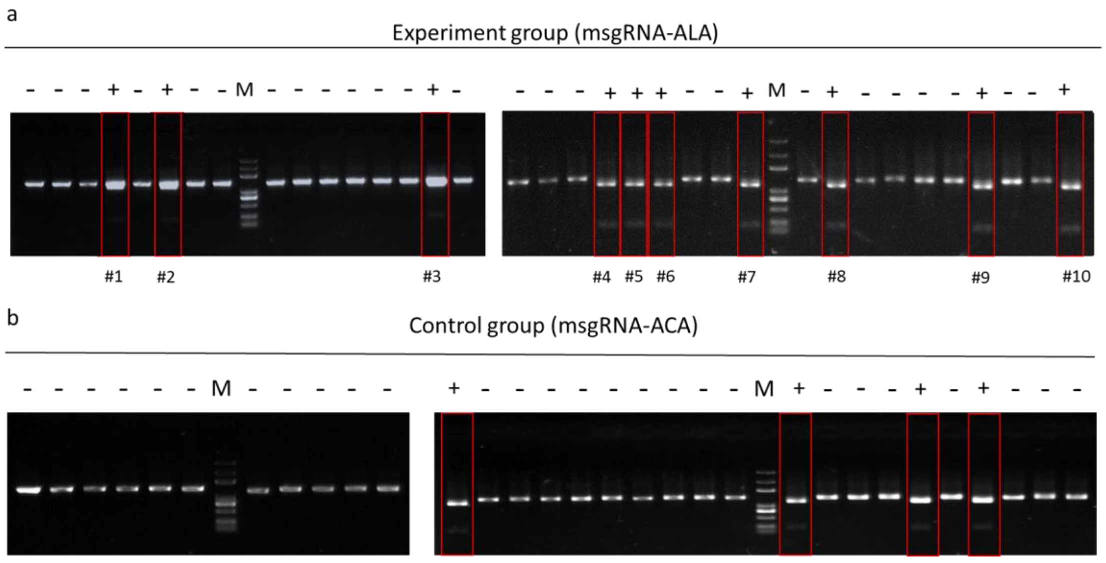

Supplementary Figure 3 The results for the detection of genome edited positive clones by *Eco*RⅠ-digesting assay. The PCR product was supposed to be 1397 bp in length and could be digested into two fragments (1194 bp and 203 bp) if the genome DNA had been successfully edited by HDR-based repair. The PCR product would be partially digested if the clone was heterozygotes with only one allele edited. Percentage of digestion positive clones within detected clones was calculated to evaluate the precise genome editing efficiency. The results demonstrated 10 positive clones out of 35 cell clones (10/35) for the msgRNA-ALA group, while 4 out of 31 clones (4/31) for the msgRNA-ACA control. However, the pictures and the bands were not clear for confirming whether one or both alleles were edited. Further repeat detection of these positive clones demonstrated that they were all heterozygotes with one allele edited (refer to Fig.4e in the manuscript).

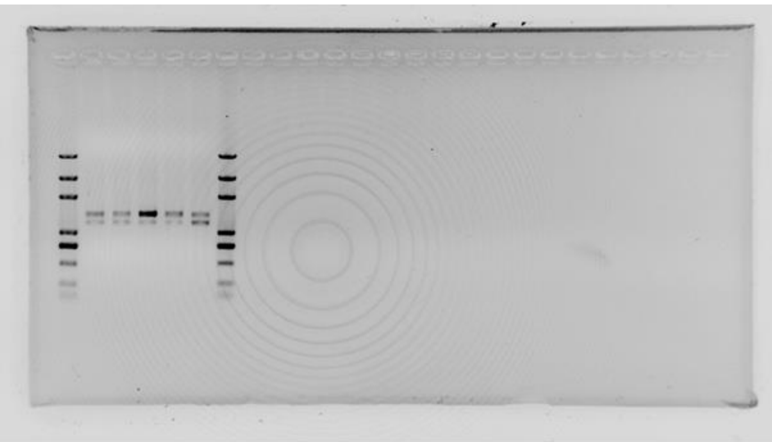

a

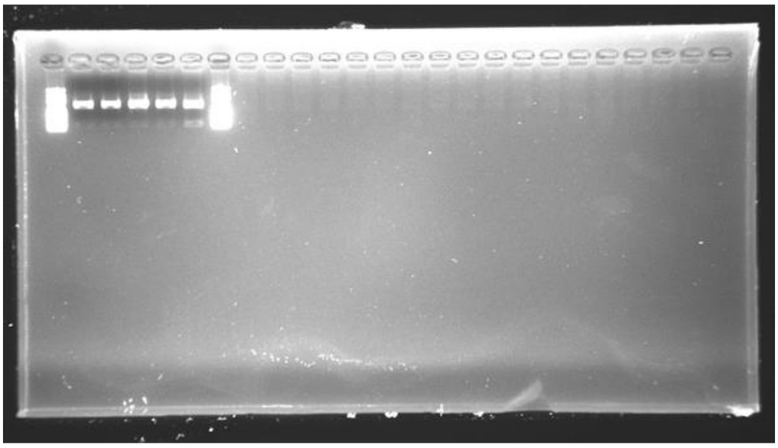

b

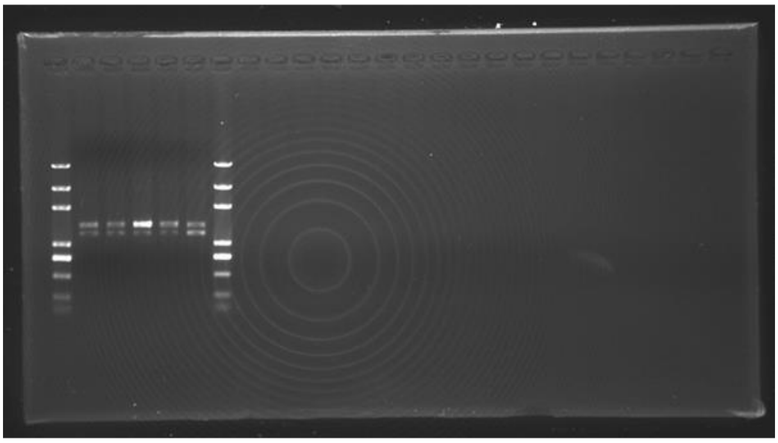

c

Supplementary Figure 4 Full-length gel images of representative results for the detection of positive clones by EcoR Ⅰ digestion. (a) Full-length gel of Fig. 4e in the manuscript. (b)

Overexposure after a shorter electrophoresis period for a clear vision of the 203bp band. (c) Normal-length exposure after a longer electrophoresis period for a clear vision of the 1397 bp and 1194 bp bands.

Supplementary Table 1 Transfection groups set for the DsRed-eGFP surrogate reporter assay

	Cas9 vector	msgRNA	Reporter	Group
Molar ratio	1	1.5	0.8	
VEGF target groups	pll3.7-U6-CMV-hStCas9	msgRNA-2	pRG.VEGF	Experiment group
	pll3.7-U6-VEGF-CMV-hStCas9	pcDNA3.1(+)	pRG.VEGF	Positive control
	pll3.7-U6-CMV-hStCas9	pcDNA3.1(+)	pRG.VEGF	Negative control
CCR5 target groups	pll3.7-U6-CMV-hStCas9	msgRNA-2	pRG.CCR5	Experiment group
	pll3.7-U6-CCR5-CMV-hStCas9	pcDNA3.1(+)	pRG.CCR5	Positive control
	pll3.7-U6-CMV-hStCas9	pcDNA3.1(+)	pRG.CCR5	Negative control

Supplementary Table 2 Transfection groups set for the multiplex genome targeting assay

	Cas9 vector	msgRNA	Reporter	Group
Molar ratio	1	1.5	0.8	
AAVS1 target	pll3.7-U6-CMV-hStCas9	msgRNA-3	pRPG.AAVS1	Experiment 1
groups	pll3.7-U6-AAVS1-CMV-hStCas9	pcDNA3.1(+)	pRPG.AAVS1	Positive control 1
VEGF target	pll3.7-U6-CMV-hStCas9	msgRNA-3	pRPG.VEGF	Experiment 2
groups	pll3.7-U6-VEGF-CMV-hStCas9	pcDNA3.1(+)	pRPG.VEGF	Positive control 2

CCR5 target	pll3.7-U6-CMV-hStCas9	msgRNA-3	pRPG.CCR5	Experiment 3
groups	pll3.7-U6-CCR5-CMV-hStCas9	pcDNA3.1(+)	pRPG.CCR5	Positive control 3

Supplementary Table 3 Diplex and triplex targeted positive clones detected in the multiplex genome targeting assay

Groups	**Number of diplex and triplex targeted positive clones/Detected cell clones**			
	AAVS1 & VEGF	VEGF & CCR5	AAVS1 & CCR5	AAVS1 & VEGF & CCR5
Experiment 1	1/12	0/12	2/12	1/12
Experiment 2	1/10	1/10	1/10	1/10
Experiment 3	0/10	0/10	1/10	1/10
General frequency	6% (2/32)	3% (1/32)	13% (4/32)	9% (3/32)

Supplementary Table 4 The overall mutated clones for each targeted locus in the multiplex genome targeting assay

Groups	**Mutated clones/Detected clones**			**Remarks**
	AAVS1	VEGF	CCR5	
Experiment 1	11/12	2/12	4/12	pRPG.AAVS1 as reporter
Experiment 2	6/10	3/10	5/10	pRPG.VEGF as reporter
Experiment 3	9/10	1/10	2/10	pRPG.CCR5 as reporter
General frequency	81% (26/32)	19% (6/32)	34% (11/32)	
Positive control 1	100% (6/6)	-	-	pRPG.AAVS1 as reporter
Positive control 2	-	100% (8/8)	-	pRPG.VEGF as reporter

Positive control 3	-	-	38% (5/13)	pRPG.CCR5 as reporter

Supplementary Table 5 Primers used in Quantitative RT-PCR assay

Primer name	Sequence	Remarks
qP-LIG4.F	GCCCGAGGCCAGTTAAACGAGAAG	For *LIG4* gene in the
qP-LIG4.R	GTGGTTCTTATGAAGAGCATCATG	qRT-PCR assay
ACTB forward	ATTGCCGACAGGATGCAGA	For *β-actin* gene in the
ACTB reverse	GAGTACTTGCGCTCAGGAGGA	qRT-PCR assay

Supplementary Sequence 1 The DNA sequence designed for the msgRNA-2 construct (between *Nhe*I and *Apa*I cutting site).

```
 NheI                                                                                                            EcoRI
gctagcctcg gccaccacag ggaagcgttt tagagctaga aatagcaagt taaaataagg ctagtccgtt atcaacttga aaaagtggca ccgagtcggt gctttgaatt
cgatcggagc cggtggtgtc ccttcgcaaa atctcgatct ttatcgttca attttattcc gatcaggcaa tagttgaact ttttcaccgt ggctcagcca cgaaacttaa
              VEGF                                          sgRNA scaffold

                                                                                                                XhoI
ccgaggcagt aggcacacac tcaaccttta tatttactac atctgtggct tcactagtaa atataaaggt tgagtgttcg ctcactgtca acagcatata ccttctcgag
ggctccgtca tccgtgtgtg agttggaaat ataaatgatg tagacaccga agtgatcatt tatatttcca actcacaagc gagtgacagt tgtcgtatat ggaagagctc
drosha cutting site                        CON. shRNA                                drosha cutting site

                                                                                                               ApaI
cacacttgtc accaccccaa gttttagagc tagaaatagc aagttaaaat aaggctagtc cgttatcaac ttgaaaaagt ggcaccgagt cggtgctttt ttgggcccgt
gtgtgaacag tggtggggtt caaaatctcg atctttatcg ttcaatttta ttccgatcag gcaatagttg aactttttca ccgtggctca gccacgaaaa aacccgggca
        CCR5                                                sgRNA scaffold
```

Supplementary Sequence 2 The DNA sequence designed for the msgRNA-3 construct (between *Sac*I and *Apa*I cut sites).

```
 SacI                                                                                                          EcoRI
gagctcctgt cccctccacc ccacaggttt tagagctaga aatagcaagt taaaataagg ctagtccgtt atcaacttga aaaagtggca ccgagtcggt gctttgaatt ccgaggcagt aggcacacac tcaaccttta
ctcgaggaca ggggaggtgg ggtgtccaaa atctcgatct ttatcgttca attttattcc gatcaggcaa tagttgaact ttttcaccgt ggctcagcca cgaaacttaa ggctccgtca tccgtgtgtg agttggaaat
             AAVS1                                                          sgRNA scaffold                                      Drosha cutting site

                                                                                       NheI
tatttactac atctgtggct tcactagtaa atataaaggt tgagtgttcg ctcactgtca acagcatata ccttgctagc ctcggccacc acagggaagc gttttagagc tagaaatagc aagttaaaat aaggctagtc
ataaatgatg tagacaccga agtgatcatt tatatttcca actcacaagc gagtgacagt tgtcgtatat ggaacgatcg gagccggtgg tgtcccttcg caaaatctcg atctttatcg ttcaatttta ttccgatcag
                       CON.shRNA                             Drosha cutting site                          VEGF                            sgRNA scaffold

                                           EcoRI
cgttatcaac ttgaaaaagt ggcaccgagt cggtgctttg aattccgagg cagtaggcac acactcaacc tttatattta ctacatctgt ggcttcacta gtaaatataa aggttgagtg ttcgctcact gtcaacagca
gcaatagttg aactttttca ccgtggctca gccacgaaac ttaaggctcc gtcatccgtg tgtgagttgg aaatataaat gatgtagaca ccgaagtgat catttatatt tccaactcac aagcgagtga cagttgtcgt
                                              Drosha cutting site                          CON.shRNA                                   Drosha cutting site

          XhoI                                                                                                                      ApaI
tataccttct cgagcacact tgtcaccacc ccaagtttta gagctagaaa tagcaagtta aaataaggct agtccgttat caacttgaaa aagtggcacc gagtcggtgc tttttgggc ccgtttaaac ccgctgatca
atatggaaga gctcgtgtga acagtggtgg ggttcaaaat ctcgatcttt atcgttcaat tttattccga tcaggcaata gttgaacttt ttcaccgtgg ctcagccacg aaaaacccg ggcaaatttg ggcgactagt
                      CCR5                                                      sgRNA scaffold
```
